# Impact of body mass index on chronic rhinosinusitis: disease burden and treatment outcomes

**DOI:** 10.1007/s00405-025-09709-x

**Published:** 2025-09-23

**Authors:** Kannanunni S., Meera N. Khadilkar, Thripthi Rai

**Affiliations:** Department of Otorhinolaryngology - Head and Neck, Kasturba Medical College Mangalore, Manipal Academy of Higher Education, Manipal, India

**Keywords:** Body mass index, Nasal polyps, Obesity, Rhinosinusitis, Waist circumference

## Abstract

**Background:**

Chronic rhinosinusitis (CRS) is a prevalent inflammatory condition that significantly affects quality of life, with obesity emerging as a key modifiable risk factor. Both body mass index (BMI) and waist circumference (WC) are associated with increased CRS prevalence, severity, and potentially poorer treatment outcomes due to sustained inflammation and altered immune responses. This study investigates how BMI and WC influence CRS severity and response to therapy.

**Methods:**

This two-year observational study included adult CRS patients, assessing BMI, WC, Rhinosinusitis Disability Index (RSDI), Sinonasal Outcome Test-22 (SNOT-22), and Lund-Kennedy endoscopic scores, before and after standard medical/ surgical treatment (3 months).

**Results:**

Of the 43 CRS patients, most showed significant symptoms and endoscopic score improvements over three months, regardless of BMI or waist circumference. While RSDI and Lund-Kennedy scores showed some group-wise differences, overall outcomes were not significantly influenced by BMI or WC categories.

**Conclusion:**

Obesity measures, as indicated by BMI, do not influence baseline CRS symptom severity but are associated with greater endoscopic improvement following treatment. All BMI groups demonstrate significant symptom relief, underscoring the effectiveness of therapy across varying body weight categories.

## Introduction

Chronic rhinosinusitis (CRS) is a common and often debilitating inflammatory condition that significantly impairs daily functioning and overall quality of life. In recent years, increasing attention has been directed toward modifiable risk factors—particularly obesity—that may contribute to both the onset and progression of CRS [1, 2].

Obesity, defined as a body mass index (BMI) ≥ 30 kg/m², is linked to a higher risk of developing CRS, with a large Norwegian population-based study reporting a 53% greater prevalence among obese individuals compared to those with normal BMI [3]. Beyond its role in disease onset, obesity may also influence CRS severity and treatment outcomes. This is thought to sustain low-grade systemic inflammation, which could exacerbate sinonasal inflammation and alter immune responses in CRS. Furthermore, obesity has been linked to glucocorticoid resistance, complicating the efficacy of corticosteroid-based therapies commonly used in CRS management [3, 4].

Central obesity, as reflected by increased waist circumference (WC), may offer additional prognostic insight. WC is a practical and clinically validated measure of visceral adiposity and is often considered a more accurate predictor of cardiometabolic risk than BMI alone [4, 5]. Elevated WC has been independently associated with comorbidities such as hypertension, diabetes, dyslipidemia, joint and back pain, and hyperuricemia [6].

Despite these associations, the specific impact of BMI and WC on CRS severity and response to medical treatment remains inadequately defined. While some studies have explored these relationships, comprehensive data on both baseline disease burden and post-treatment outcomes across BMI and WC strata are limited. This study aims to assess the influence of BMI and WC on the baseline severity of CRS—as measured by symptom scores, endoscopic findings, and radiological assessments—and to evaluate changes in these parameters following standard medical therapy. By examining these associations, the study seeks to contribute to the growing body of evidence that supports individualized management strategies for patients with obesity-related CRS, potentially informing both clinical care and public health interventions [2, 7, 8].

## Methods

A two-year observational study was conducted on patients aged 18 to 60 years, of both genders, diagnosed with chronic rhinosinusitis (CRS) based on criteria from the European Position Paper on Rhinosinusitis and Nasal Polyps 2012 (EPOS 2012) and the American Academy of Otolaryngology. Exclusion criteria included previous nasal or paranasal sinus surgery, head and neck malignancy, unwillingness to undergo required investigations or treatment, and inability to complete a minimum of three months’ follow-up. The sample size of 43 was calculated using the prevalence-based formula by Lemeshow et al. (1990) [9]. A non-random convenience sampling method was used. All eligible participants provided informed consent and underwent demographic profiling, medical history documentation, and thorough otolaryngology examination.

Clinical assessment included the measurement of BMI, WC, Rhinosinusitis Disability Index (RSDI), Sinonasal Outcome Test-22 (SNOT-22), and Lund-Kennedy endoscopic scores, recorded at baseline and re-evaluated one- and three-months post-treatment. Initial medical therapy for all patients comprised amoxicillin–clavulanate (40 mg/kg/day of amoxicillin in three divided doses) for two weeks, and cetirizine 5–10 mg once daily. Patients with comorbid allergic rhinitis additionally received intranasal fluticasone furoate (110 mcg/day) for one month and oral deflazacort in tapering doses (starting at 0.9 mg/kg/day). Patients unresponsive to medical therapy were managed surgically based on clinical indications, including endoscopic sinus surgery or septoturbinoplasty. Statistical analysis was performed using SPSS version 25, with significance set at *p* < 0.05.

## Results

A total of 43 patients of CRS were included in the study (of which 14 had polyps), with 31 undergoing both medical and surgical Management and 12 receiving medical therapy alone. Participants ranged in age from 21 to 60 years (Table 1), with a trend of increasing overweight and obesity prevalence in the older age groups. The mean height was 163.6 cm, mean weight 68.9 kg, and mean waist circumference 88.8 cm, with a Maximum of 102 cm, indicating a risk of central obesity.

**Table 1:** Distribution of variables

Age (years)	BMI	*N* (%)			
21–30	18.6–24.9	7 (16.3%)			
	25-29.9	3 (7.0%)			
	> 30	1 (2.3%)			
31–40	18.6–24.9	3 (7.0%)			
	25-29.9	11 (25.6%)			
	> 30	1 (2.3%)			
41–50	18.6–24.9	4 (9.3%)			
	25-29.9	1 (2.3%)			
	> 30	1 (2.3%)			
51–60	18.6–24.9	4 (9.3%)			
	25-29.9	6 (14.0%)			
	> 30	1 (2.3%)			
**Variable**		**N**		**Mean ± SD**	**Median**
Age	18.6–24.9 BMI	18	37.8 ± 12.8		31.5
	25-29.9 BMI	21	41.2 ± 10.8		39
	> 30 BMI	4	42.3 ± 9.98		43
Height (m)		43		163.6 ± 8.19	165
Weight (kg)		43		68.9 ± 10.14	68
Waist Circumference (cm)		43		88.8 ± 6.6	91.4

*SD* standard deviation

RSDI scores showed a significant difference between BMI groups at baseline (*p* = 0.036) but not at follow-up (*p* = 0.299), with all groups improving significantly over time (*p* < 0.001) (Table 2). SNOT-22 scores showed no between-group differences at baseline (*p* = 0.209) or follow-up (*p* = 0.333), but each group improved significantly (*p* < 0.001). Lund-Kennedy scores showed no baseline difference (*p* = 0.837) but differed significantly between groups at three months (*p* = 0.049), with all groups showing improvement (*p* < 0.001). Modified Lund-Kennedy scores showed no significant between-group differences at baseline (*p* = 0.766) or follow-up (*p* = 0.493), though all BMI groups demonstrated significant improvement from baseline (*p* < 0.001).

**Table 2:** Baseline and follow-up scores across BMI categories

Score	BMI Category	Baseline (Mean ± SD)	Follow-up (Mean ± SD)	*p*-value (Timeline)	*p*-value (Baseline)	*p*-value (Follow-up)
RSDI (0–120)	18.6–24.9	59.7 ± 7.17	43.9 ± 5.35	< 0.001*	0.036*	0.299
	25–29.9	63.6 ± 8.00	43.4 ± 5.69	< 0.001*		
	> 30	69.5 ± 5.26	49.8 ± 6.85	< 0.001*		
SNOT-22 (0–110)	18.6–24.9	56.5 ± 12.08	40.7 ± 6.81	< 0.001*	0.209	0.333
	25–29.9	61.0 ± 9.00	40.9 ± 8.04	< 0.001*		
	> 30	66.3 ± 8.18	49.2 ± 9.95	< 0.001*		
Lund-Kennedy (0–20)	18.6–24.9	9.83 ± 2.04	4.61 ± 1.34	< 0.001*	0.837	0.049*
	25–29.9	10.0 ± 1.64	4.76 ± 0.99	< 0.001*		
	> 30	10.5 ± 1.92	6.25 ± 0.96	< 0.001*		
Modified Lund-Kennedy (0–12)	18.6–24.9	6.44 ± 1.58	2.80 ± 0.84	< 0.001*	0.766	0.493
	25–29.9	6.71 ± 1.42	2.60 ± 0.55	< 0.001*		
	> 30	7.00 ± 1.41	3.00 ± 0.00	< 0.001*		

*SD* standard deviation

Waist circumference (WC) categories (< 90 cm vs. ≥ 90 cm) showed no significant differences at baseline across RSDI, SNOT-22, Lund-Kennedy, or modified Lund-Kennedy scores (Table 3); however, all outcome measures demonstrated significant within-group improvements at three-month follow-up (*p* < 0.001).

**Table 3:** Comparison of CRS scores by waist circumference (WC) in women and men

Score	WC Group	Baseline (Mean)	Follow-up (Mean)	*p*-value (Timeline)	*p*-value (Baseline)
Women					
RSDI (0–120)	< 80 cm	51	40	< 0.001*	0.635
	≥ 80 cm	62.1	46.5	< 0.001*	
SNOT-22 (0–110)	< 80 cm	54	39.5	< 0.001*	0.639
	≥ 80 cm	59.4	42.1	< 0.001*	
Lund-Kennedy (0–20)	< 80 cm	8	3	< 0.001*	0.982
	≥ 80 cm	10.11	5.11	< 0.001*	
Modified Lund- Kennedy (0–12)	< 80 cm	5	2.33	< 0.001*	0.456
	≥ 80 cm	6.74	2.83	< 0.001*	
Men					
RSDI (0–120)	< 90 cm	58.5	44	< 0.001*	0.639
	≥ 90 cm	63.8	46.8	< 0.001*	
SNOT-22 (0–110)	< 90 cm	54.5	46	0.003*	0.639
	≥ 90 cm	60.5	42.3	< 0.001*	
Lund-Kennedy (0–20)	< 90 cm	11	4.5	< 0.001*	0.982
	≥ 90 cm	9.86	4.71	< 0.001*	
Modified Lund- Kennedy (0–12)	< 90 cm	7.5	3	< 0.001*	0.635
	≥ 90 cm	6.52	2.6	< 0.001*	

*statistically significant

There was no significant association between BMI or WC categories and baseline Lund-Mackay scores (p = 0.429 and p = 0.0529, respectively) (Table 4).

**Table 4:** Comparison of Lund-Mackay scores with BMI and WC

Group	Category	*N*	Mean Score	SD	*p*-value
BMI	18.6–24.9	18	10.8	2.15	0.429
	25–29.9	21	11.5	2.14	
	> 30	4	12.0	1.41	
WC	< 90 cm	21	11.0	2.11	0.529
	≥ 90 cm	22	11.5	2.09	

*SD *standard deviation

## Discussion

Recent evidence suggests that obesity, measured by BMI, may affect both the severity and treatment outcomes of chronic rhinosinusitis (CRS), possibly due to increased systemic and local inflammation [3]. In this study, individuals aged 21–30 mostly had normal BMI, while higher BMI categories were more common in older age groups (31–40 and 51–60), reflecting known epidemiological patterns. Waist circumference (WC)—a measure of central obesity defined as > 90 cm in men and > 80 cm in women [6]—also tended to be elevated among older participants and those with higher BMI, with an average WC of 88.8 cm and a Maximum of 102 cm observed. Given its association with cardiometabolic risk and systemic inflammation [6], WC was included as a relevant clinical parameter.

A significant association was found between BMI and baseline RSDI scores (*p* = 0.036), indicating that individuals with higher BMI reported a greater perceived symptom burden. However, this difference was not evident after one month (*p* = 0.299), suggesting comparable short-term symptom relief across BMI groups. Similarly, SNOT-22 scores showed no significant differences at baseline (*p* = 0.209) or one-month follow-up (*p* = 0.333), consistent with previous reports by Clarhed et al. and Sreeparvathi et al. that obesity increases the risk and baseline impact of CRS but does not impair treatment response [3, 10]. Lund-Kennedy endoscopic scores did not differ at baseline (*p* = 0.837), but a significant difference appeared after three months (*p* = 0.049), indicating that BMI may affect mucosal healing over time, likely through persistent low-grade inflammation [3, 10, 11]. In contrast, modified Lund-Kennedy scores showed no baseline or intergroup differences, although all BMI categories demonstrated significant post-treatment improvement (*p* < 0.001), in line with findings by Steele et al. [12]. BMI plays a complex role in CRS, influencing both baseline symptom perception and long-term mucosal recovery, without altering the trajectory of early clinical improvement.

Regarding central obesity, no significant baseline differences were observed in symptom or endoscopic scores between participants with and without elevated WC, indicating that central obesity may not influence initial CRS severity. However, participants with higher WC showed greater improvement in both Lund-Kennedy and modified scores (*p* < 0.001), suggesting a role in mucosal recovery. This mirrors the findings of Habib et al. and Clarhed et al., who reported links between central obesity and the CRS with nasal polyps (CRSwNP) phenotype, particularly with symptoms such as olfactory loss and purulence. These symptoms, however, were not accompanied by a corresponding reduction in treatment response [3, 13]. Adipose tissue is known to influence blood flow and inflammation [14], an aspect not directly evaluated in the present study. As a result, central obesity may shape both the course of the disease and the pace of recovery, without necessarily making the initial symptoms more severe. Further studies are needed to validate potential mechanisms related to tissue perfusion and corticosteroid responsiveness in the context of central obesity.

Baseline Lund-Kennedy and Lund-Mackay scores did not significantly differ across BMI (*p* = 0.429) or WC groups (*p* = 0.0529), indicating that neither general nor central obesity correlates with initial objective CRS severity. This agrees with previous studies showing that while obesity elevates CRS risk, it does not necessarily reflect in baseline imaging or endoscopic burden [10, 15]. The Lund-Kennedy score remains a reliable severity marker, consistent with its established correlation with the Lund-Mackay CT score and clinical outcomes [10, 15]. While obesity may be linked to systemic inflammation and glucocorticoid resistance, particularly in eosinophilic CRS [11], studies like DeConde et al. have reported similar baseline disease severity and quality-of-life measures across BMI groups despite higher comorbidities [10, 12]. Likewise, no radiological differences across WC groups were seen, reinforcing evidence from Habib et al. and Nam et al. that central obesity, though more common in CRSwNP, does not correlate with radiographic severity or symptom burden in CRS without nasal polyps. Mendelian randomization studies also support the absence of a causal link between central obesity and radiological disease burden [13, 16].

Overall, the findings underline the multifaceted role of obesity in CRS. While higher BMI and WC may influence symptom perception and long-term mucosal response, they do not significantly alter short-term therapeutic benefit. This supports the need for individualized treatment strategies, especially for overweight patients who may experience different inflammatory or healing profiles with therapies like glucocorticoids or biologics. Tailored care, lifestyle modification, and multidisciplinary collaboration remain critical. However, the current study’s limitations—including small subgroup sizes, brief follow-up, and lack of control for confounding variables such as smoking, asthma, or metabolic markers—highlight the need for broader, long-term research to fully understand the implications of obesity on CRS course and outcomes.

## Conclusion

Obesity and central adiposity are associated with increased risk and symptom burden in chronic rhinosinusitis, without influencing baseline endoscopic or radiologic severity or limiting short-term treatment response. Meaningful improvement occurs across all body mass index and waist circumference groups with appropriate therapy, supporting the importance of individualized care and weight management. Further large-scale, long-term research helps clarify the role of obesity in chronic rhinosinusitis progression and treatment outcomes.
